# Optimization of Bacterial Plasmid Transformation Using Nanomaterials Based on the Yoshida Effect

**DOI:** 10.3390/ijms11124962

**Published:** 2010-12-03

**Authors:** Haidong Tan, Li Fu, Masaharu Seno

**Affiliations:** 1 Biotechnology Department, Dalian Institute of Chemical Physics, Chinese Academy of Sciences, Dalian 116023, China; E-Mail: hdt@dicp.ac.cn; 2 Department of Breast Cancer Pathology and Research Laboratory, State Key Laboratory of Breast Cancer Research, Cancer Hospital of Tianjin Medical University, Tianjin 300060, China; E-Mail: fulijyb@hotmail.com; 3 Department of Medical and Bioengineering Science, Graduate School of Natural Science and Technology, Okayama University, Okayama 700-8530, Japan

**Keywords:** sepiolite, carbon nanotube, plasmid transformation, nanomaterials, Yoshida effect

## Abstract

With the help of sepiolite, a unique method for transforming DNA into bacteria, based on the Yoshida effect, has been developed recently. However, we confronted many problems when this newest method was tried. Only a few transformants could be obtained even when 100 ng of plasmid pET15b was used, and a successful result seemed difficult to repeat. To address this problem, we optimized the operating method and could achieve about 15,000 transformants using the same amount of plasmid, which could match the efficiency gained using the calcium chloride transformation method. Meanwhile, the results could also be reproduced well. In the same way, carbon nanotubes were used to attain more than 15,000 transformants in the same situation. Therefore, the transformation method could be extended to other nanomaterials. Meanwhile, compared with the mechanism previously reported, we verified quite a different principle for the mechanism responsible for such a transformation. In sum, this unique transformation can be developed to become the third widely-used transformation method in laboratories in addition to the chemical method and electroporation.

## 1. Introduction

In 2001, Yoshida and colleagues published a novel transformation method based on the inoculation of transforming DNA into bacteria by means of mineral nanofiber [1]. However, little notice has been taken of this very interesting invention because (i) the published work referred to the use of chrysotile asbestos fibers, which had carcinogenic potential and biological activity [2]; and (ii) the authors suggest the usage of a specific apparatus for optimized application of sliding friction forces, which could discourage possible users [3]. In 2010, Wilharm and colleagues improved the method based on the Yoshida effect using another mineral nanofiber (sepiolite) [4], which had been introduced by Yoshida and Sato [5]. Sepiolite—an inexpensive, resourceful, fibrous yet inoffensive mineral—makes DNA transformation rapid and simple. However, we confronted many problems using the novel method: The result seemed difficult to repeat and only a few tranformants could be obtained even when 100 ng of plasmids were used. In this case, we optimized the protocol, but it was difficult to attain more than 3,000 of transformants when 100 ng plasmid pET15b was used.

To address this problem, we changed the operating method and thereby enhanced the transformation efficiency greatly, which could match with that gained from chemical method. We guessed that such a transformation could be extended to other nanomaterials. For example, carbon nanotubes (CNTs) are widely used in most labs and are well characterized [6]. CNTs have been reported as a DNA carrier during the electroporation [7]; however the results cannot point to CNTs making plasmid transformation occur, so the utilization of CNTs for DNA transformation is still unreported. Using our new method, we tried plasmid transformation based on CNTs and got more than 15,000 transformants for 100 ng pET15b.

From these results and combining with those of other experiments, we found that the present mechanism could not explain our findings, so an alternative mechanism was provided here. Meanwhile, with more and more nanomaterials being invented, we hope this unique transformation can be developed to be a third widely-use method in most labs in addition to the chemical method and electroporation.

## 2. Results and Discussion

### 2.1. Optimization of Parameters for Plasmid Transformation by Means of Sepiolite

**Figure 1.**
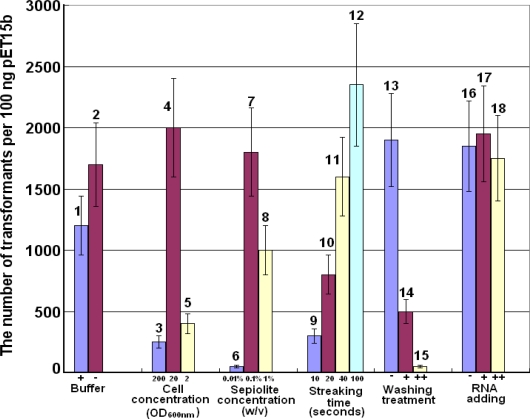
The optimization of DNA transformation for *Escherichia coli* DH5α based on sepiolite. (**1**–**2**) The effect of sepiolite buffer on transformation frequency: Sepiolite buffer was used in sample 1 and LB medium was used directly in sample 2; (**3**–**5**) The effect of varying cell concentration: OD_600 nm_ = 200, 20 and 2 in sample 3, 4 and 5 respectively; (**6**–**8**) The effect of percent content of sepiolite: 1%, 0.1% and 0.01% in sample 6, 7 and 8 respectively; (**9**–**12**) The effect of time of streaking to plate the transformed cells: 10, 20, 40 and 100 s in sample 9, 10, 11 and 12 respectively; (**13**–**15**) The effect of washing treatment with ddH_2_O on transformation frequency. Sample 13, DNA-binding sepiolite. The DNA-binding sepiolite was washed once and twice by ddH_2_O in sample 14 and 15; (**16**–**18**) The effect of RNA competition on transformation frequency. Sample 16, no RNA was added. For sample 17 and 18, 300 ng and 3 μg small RNA was added in 50 μL transforming solution.

We confronted many problems when we repeated the procedure provided in a previous report [4], and only a few transformants could be obtained although 100 ng of plasmid pET15b was used (Figure 1, sample 6). Moreover, the work was challenging to repeat. Therefore, we optimized various parameters for such a transformation method. We found that the sepiolite buffer was not necessary since the result was no better that when LB medium was used (Figure 1, samples 1 and 2). Furthermore, the cultured cells could be transformed by plasmids in LB medium directly. On the other hand, 0.01% sepiolite was too low to be successful for such a transformation (Figure 1, samples 6–8). Additionally, the time of streaking the plate to spread the transformed cells could affect the efficiency of plasmid transformation greatly (Figure 1, samples 9–12).

### 2.2. Plasmid Transformation by Means of Sepiolite and Vortex Operation

**Figure 2.**
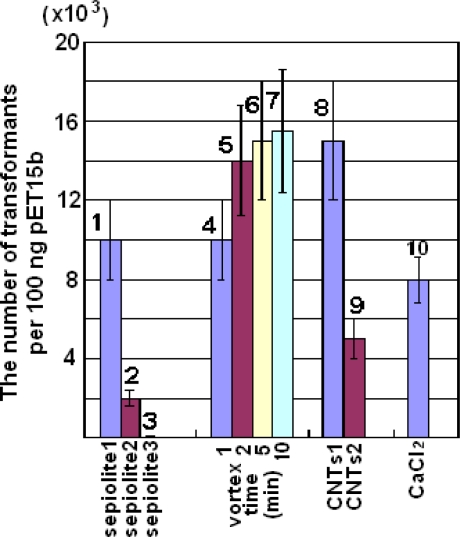
DNA transformation for *Escherichia coli* DH5α based on sepiolite and vortex mixing. (**1**–**3**) Untreated sepiolite (sepiolite 1), the sepiolite collected from the supernatants (sepiolite 2) and ultrasonicated sepiolite (sepiolite 3); (**4**–**7**) Effect of varying vortex operation time for sepiolite 1, from 1 to 10 min; (**8**–**9**) Untreated CNTs (CNTs1) and ultrasonicated CNTs (CNTs2). CaCl_2_ refers to chemical transformation method.

Even after the optimization mentioned above, high-efficiency transformation was still difficult to achieve. We decided to repeat the work using a vortex since all parameters could be more easily controlled in a microtube. Thus, we repeated the plasmid transformation with vortexing the mixture (plasmid, sepiolite and cell in LB medium) vigorously in a microtube. Just as we expected, 15,000 transformants per 100 μg of pET15b could be attained, which matched the efficiency obtained from the calcium chloride transformation (8,000 transformants per 100 ng of pET15b) (Figure 2, samples 4–7 and 10). For the operation, one point should be considered carefully. The transformation efficiency was reduced greatly when the volume of the mixture was more than 100 μL in a 500-μL microtube. We also found the efficiency increased when the vortex time was prolonged. However, the enhancement was slight for vortexing times longer than 5 min although there was no trend for the transformation efficiency reducing with time (Figure 2). Additionally, longer vortex time had no detrimental effect on cell viability or morphology.

### 2.3. Plasmid Transformation by Means of CNTs

Since the plasmid transformation performed well using nanofibers, the method should be extended to other nanomaterials. For this purpose, we used CNTs in the transformation and obtained more than 15,000 transformants per 100 ng pET15b, which was more than we obtained using a traditional chemical method (Figure 2, samples 8 and 10). However, the result could not be explained by the fibers causing piercing of the bacterial cell wall, because we observed that many CNTs were congregated together under the microscope while the ultrasonicated CNTs failed to produce more transformants (Figure 2, sample 9). To identify an alternative explanation for the mechanism of the transformation, the work described below was performed.

### 2.4. Mechanism for the Plasmid Transformation by Means of Nanomaterials

Previously, the mechanism for the transformation method based on the inoculation of transforming DNA into bacteria by means of mineral nanofiber was explained as follows: When a colloidal solution containing mineral nanofibers is mixed with bacteria and transforming DNA and plated on selective agar plates, the sliding friction forces, arising between the surface of the agar and the stir stick when bacteria are spread, result in penetration of bacterial cells, which leads to inoculation of the transforming DNA that is adsorbed to the mineral nanofibers. Inside the cell, the DNA is probably displaced by competition with small nucleic acids [4], so that it can be maintained and expressed. According to this mechanism, it was obvious that the transformation efficiency would be reduced greatly if enough small RNA was added to the mixture because this transformation with exogenous DNA was closely related to the phenomenon based on the Yoshida effect. To test the hypothesis, the following experiments were prepared.

**Figure 3.**
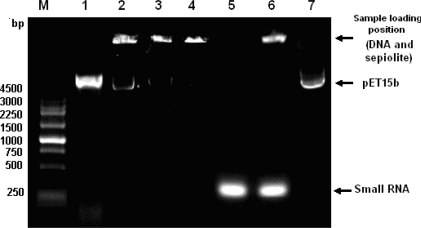
1% agarose analysis of DNA-binding after sepiolite washing and competitive absorbtion with small RNA. **Lane 1**: pET15b expression vector (∼5.3 Kb); **Lane 2**: DNA binding sepiolite to saturation; **Lane 3**: DNA binding sepiolite to saturation was washed once with water; **Lane 4**: DNA binding sepiolite to saturation was washed with water twice; **Lane 5**: Small RNA (∼300 bp) extracted from *Lactobacillus brevis* AS1.579; **Lane 6**: The mixture of DNA-binding sepiolite and small RNA; **Lane 7**: DNA-binding sepiolite was eluted by 400 mM guanidine hydrochloride, pH 4.0. Note: After electrophoresis, the DNA stayed at the sample loading position due to the strong absorbing power between the DNA and sepiolite (**Lanes 2**–**4**, **6**).

Firstly, as shown in Figure 3, lane 5, high-quality 300-bp RNA (300 ng/μL) was extracted from *Lactobacillus brevis* AS1.579 and prepared according to the method described in a previous report [8], and briefly in the Experimental Section. Through a competitive absorbtion test, we found that the DNA on the sepiolite could not be replaced by 300 ng/μL RNA (Figure 3, lane 6). The DNA could not even be eluted out by 5 M NaCl (data not show), but could be eluted out by 400 mM guanidine hydrochloride, pH 4.0 (Figure 3, lane 7). This result indicated that the DNA absorbed sepiolite tightly and could not be displaced by RNA. On the other hand, washing treatment with ddH_2_O could affect plasmid transformation greatly (Figure 1, samples 13–15). All together, these results implied that it was unlikely that the mechanism for the plasmid transformation was mainly caused by sepiolite carrying the DNA into cells and being displaced by RNA. The number of the transformants was mainly determined by the amount of DNA in the solution but not determined by the amount of the DNA bound on the sepiolite.

To further investigate this, we performed the following experiment. The sepiolite was absorbed to saturation with the RNA (after centrifugation, the OD_260 nm_ value of the supernatant was not reduced any more for 0.1% sepiolite solution (w/v) when more than 6 ng/μL final concentration RNA was added) and then mixed with suitable amounts of the plasmids. The result indicated the small RNA did not affect the DNA transforming frequency compared to the sample without RNA addition (Figure 1, samples 16–18). Thus, small RNA did not inhibit the DNA transformation frequency; namely, the DNA absorbed on the nanofibers would not be displaced by competition with small nucleic acids in the cell [4].

**Figure 4.**
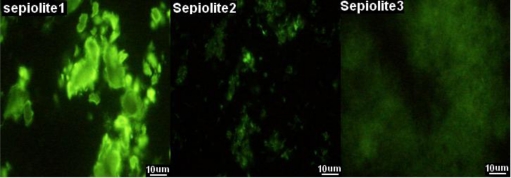
After absorbing GFPuv, the shape of sepiolite is observed by fluorescence microscope. There are three kinds of sepiolite: untreated sepiolite (sepiolite 1), the sepiolite collected from the supernatants (sepiolite 2) and ultrasonicated sepiolite (sepiolite 3).

Additionally, we selected two other sepiolites (sepiolite 2 and 3) with more fibers compared with sepiolite 1 (the untreated sepiolite), see Figure 4. Sepiolite 2 is the sepiolite collected from the supernatants and sepiolite 3 is ultrasonicated sepiolite (Figure 4). According to the mechanism supplied by previous reports [4], sepiolite with more nanofibers will produce higher-efficiency transformation. Conversely, the transformation efficiency was reduced greatly, and even failed to produce transformants when ultrasonicated sepiolite was used, while the transformation efficiency was the highest when the sepiolite was untreated (Figure 2, samples 1–3). In the same way, using CNTs produced similar results. These results suggested that the fibers alone hardly influenced the success of the plasmid transformation. Heterogeneous DNA still could enter the cell even without absorbing on the sepiolite fibers. The transformation might need the larger size of sepiolite to destroy the cell wall for the plasmids to enter the cell envelope through the temporary nanochannels formed in the cell membrane.

The most widely used methods for DNA transformation of eukaryotes are chemical transformation and electroporation. However, both methods require preparation of competent cells and recovery, which are time-consuming and tedious. Furthermore, existing electroporation technology is limited in its ability to treat large quantities of cells and DNA. Additionally, the application of an expensive electroporation apparatus and a specialized power supply can lead to irreversible electroporation and, consequently, cell lysis [9]. In some routine experiments, high efficiency is as important as time and convenience. Thus, in 2001, Yoshida and colleagues published many exciting papers describing a novel transformation method based on mineral nanofibers [10–13]. In 2010, Wilharm *et al*. further improved the method [4].

**Figure 5.**
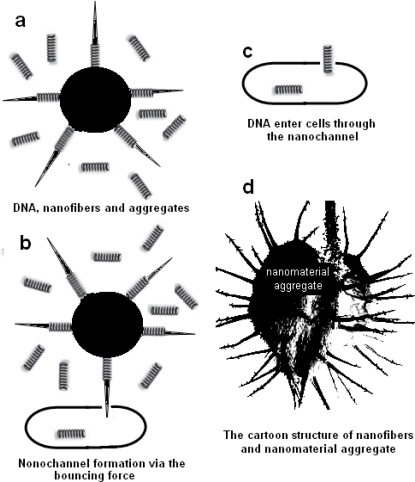
The hypothesis for the mechanism of plasmid transformation by means of nanomaterials. (**a**–**c**) The model for the process of heterogeneous DNA entering a cell with the help of nanomaterials; (**d**) the cartoon structure of nanofiber and nanomaterial aggregates.

However, we found that this latest technology still needed optimization to be widely used in most labs because we obtained low-efficiency transformation. Therefore, we optimized the protocol and changed the operation. Following our optimization, high-efficiency transformation could be reached and the results could be repeated well. In addition, through our work, we found that the present mechanism proposed to explain the transformation could not explain our results. From our results, we propose that the mechanism for the DNA transformation based on sepiolite might be explained as follows: the formation of nanochannels is driven by nanomaterials through the bouncing forces arising among the nanomaterials, bacteria and microtubes during the vortex mixing step. The heterogeneous DNA can simultaneously enter the cells via the nanopores, so that it can be maintained and expressed. To produce enough bouncing forces, the nanofibers should be fixed on the large particles of the nanomaterial aggregates (Figure 5d). Otherwise, only nano-size fibers failed to produce transformants (Figure 4 and Figure 2, sample 3).

In any way, based on nanomaterials, the transformation offer several advantages over the chemical method and electroporation: (1) The experiment can be performed conveniently at any time. For instance, DNA transformation will occur just by shaking when the sepiolite is mixed with heterogeneous DNA and bacteria in a microtube; (2) small amount of cells can be transformed by plasmids easily. For example, a single colony can be transformed by plasmids directly even when the colony has been stored on a plate at 4 °C for more than one month; (3) the experiment for the reception of some pathogens to various plasmids can be performed easily based on nanomaterials; (4) with the help of the plasmid pKD46, linear DNA can be transferred into bacteria; work which always fails via the chemical method. With more invent of nanomaterials, there are still many possibilities for the development of the transformation method. This method should be developed to become a third widely-used method in addition to the electroporation and chemical transformation methods.

On the other hand, the following work is needed to be done in the future. The method is never used for eukaryote transformation and special species. For example, electroporation and the chemical transformation method seem to be difficult to use in the areas where cell surfaces are hard, such as Gram positive bacteria or plant cells. In these cases, the plasmid transformation by means of nanomaterials may be a good choice.

## 3. Experimental Section

### 3.1. Materials and Strains

Plasmid pET15b was purchased from Novagen (Merck KGaA, Darmstadt, Germany). *Escherichia coli* DH5α, cell culture components, guanidine thiocyanate, phenol, proteinase K and plasmid minipreparation kits were supplied by Dingguo Biotech (Beijing, China). *Lactobacillus brevis* AS1.579 was obtained from The Institute of Microbiology, Chinese Academy of Sciences (Beijing, China). Sepiolite was purchased from Kremer Pigmente GmbH & Co. KG (Hauptstr, Aichstetten, Germany). Carbon nanotube (95%, 8–15 nm) was purchased from TimesNano (Chengdu, China). Recombinant green fluorescent protein (GFPuv) was expressed from *Escherichia coli* BL21 and purified in our lab.

### 3.2. Cell Culture

*Escherichia coli* DH5α was streaked and grown at 37 °C on Luria-Bertani (LB) plates (10 g/L tryptone, 5 g/L yeast extract, 10 g/L sodium chloride and 1.5% agarose, pH 7.0) for 20 h. One colony from the plate was transferred into 5 mL of LB medium, incubated at 37 °C, 200 rpm for 12 h. The pre-culture was diluted 100-fold to inoculate 500 mL into LB medium. The culture was allowed to grow with shaking (200 rpm) at 37 °C until the OD_600 nm_ reached 0.4. The cells were collected via centrifugation at 4 °C for 10 min at 3,000 × g. All the precipitation was resuspended in 1 mL sepiolite buffer, and stored on ice for the next step.

### 3.3. Optimization of Parameters for Plasmid Transformation by Means of Sepiolite

The following parameters were optimized: sepiolite buffer, cell concentration, sepiolite concentration, and the length of time spent streaking the cells on plates after transformation (Figure 1). Sepiolite buffer was prepared as previous reported [4]. One hundred microliters of transforming mixture consisting of 0.1% sepiolite (w/v), bacteria (OD_600 nm_ = 20) and 100 ng pET15b were spread on a 2% agar plate with 100 μg/mL ampicillin unless otherwise indicated. The suspension was spread with polystyrene or glass stir sticks until the liquid was completely soaked into the agar, which is indicated by a marked increase of frictional resistance, and spreading was then continued for an additional 30 s unless otherwise indicated. Plates were incubated at 37 °C overnight.

### 3.4. Plasmid Transformation by Means of Sepiolite and Vortex Operation

Fifty microliters of transforming mixture consisting of LB, bacteria (OD_600 nm_ = 20), 0.1% sepiolite and 100 ng of DNA were transferred into a 500-μL microtube. The mixture was subjected to a vortex mixer (Vortex-Genie 2, Model G560E; Scientific Industries, Inc. Bohemia, New York, USA) at full speed for 1, 2, 5 or 10 min. The vortexed cells were spread on a 1.5% agar plate with 100 μg/mL ampicillin and incubated at 37 °C overnight.

### 3.5. Plasmid Transformation by Means of CNTs

Fifty microliters of transforming mixture consisting of LB, bacteria (OD_600 nm_ = 20), 0.1% CNTs and 100 ng pET15b was transferred into a 500-μL microtube. The mixture was subjected to a vortex mixer at full speed for 1 min. The cells were then spread on a 1% agar plate with selective antibiotics (100 μg/mL ampicillin) and incubated at 37 °C overnight.

### 3.6. Plasmid Transformation by the Calcium Chloride Method

*Escherichia coli* cells were made competent for plasmid transformation by the calcium chloride method [14]. One hundred microliters of competent cells were taken and 100 ng pET15b were added. The mixture was incubated on ice for 30 min and then heat shocked at 42 °C for 90 s. The transformation mixture was incubated on ice for 2 min. Four hundred microliters of LB medium was added and the mixture incubated at 37 °C for 45 min in an incubator-shaker. Fifty microliters of the mixture was spread onto a LB plate with ampicillin and incubated at 37 °C overnight.

### 3.7. Mechanism for the Plasmid Transformation by Means of Nanomaterials

Theoretically, the DNA bound with sepiolite will not be eluted by ddH_2_O because of the strong binding force between the negative charges of DNA and a positive charge cluster of sepiolite. Thus, the amount of DNA in the solution of sepiolite will not affect the number of transformants according to the mechanism supplied by Wilharm [4]. To prove this, we removed the DNA in the solution of sepiolite via washing treatment. One hundred microliters of 0.1% sepiolite suspension (w/v) was mixed with 100 ng pET15b and then centrifuged at 5,000 × g for 2 min. The precipitate was resuspended in 500 μL ddH_2_O and the above steps were repeated twice. Finally, the precipitation of sepiolite was resuspended in 90 μL ddH_2_O and mixed with 10 μL cells (OD_600 nm_ = 200) in a 500-μL microtube. The mixture was mixed in a vortex mixer at full speed for 1 min. After mixing in a vortex, the cells were spread on a 1% agar plate with 100 μg/mL ampicillin and incubated at 37 °C overnight.

Small RNA (∼300 bp) was prepared from 10 mL late-exponential-phase *Lactobacillus brevis* AS1.579 using the CTAB preparative protocol for bacterial genomic DNA isolation, as described previously with some modification [8] and no RNAase was added. To culture *Lactobacillus brevis* AS1.579, the initial inoculum concentration was about 0.1 OD_600 nm_ in 10 mL MRS medium, and cells were grown at 37 °C for 4 h to reach an optical density at 0.8 OD_600 nm_. Basically, for RNA isolation, the cells were collected from 10 mL cultures by centrifugation at 4,000 × g for 5 min at 4 °C. A guanidine thiocyanate method was modified by adding a pre-warm step of lysis buffer. In brief, the cells were washed once by 1 mL prechilled PBS (137 mM NaCl, 2.7 mM KCl, 10 mM Na_2_HPO_4_, 2 mM KH_2_PO_4_, pH 7.4). Cells were suspended in 500 μL TE buffer (10 mM Tris-HCl, 1 mM EDTA, 10 mM lysozyme, 10 mM DTT, and 37 °C) for 30 min. Fifty units of proteinase K was added and incubated at 65 °C for 30 min. 100 μL 5 M NaCl, 100 μL CTAB was added and incubated at 65 °C for 10 min. Finally, 500 μL phenol/chloroform/isoamyl alcohol (24:24:1, v/v/v, pre-warmed to 60 °C) were added orderly and mixed. The mixture was vortexed vigorously for 30 s and cooled on ice for 15 min, followed by centrifugation at 15,000 × g for 10 min at 4 °C. To precipitate RNA, the aqueous phase was transferred to a 1.5 mL RNase-free Eppendorf tube, mixed with an equal volume pre-chilled isopropanol and stored at −20 °C for 2 h. The RNA pellet was collected by centrifugation at 10,000 × g for 15 min at 4 °C and washed twice with 75% (v/v) ethanol, air dried and dissolved in 10 μL RNase-free water. The isolated small RNA was viewed using 1% agarose gel electrophoresis.

Following the washing treatment, the competitive absorbtion for sepiolite between DNA and RNA was tested. Ten milligrams precipitated sepiolite was added to 10 μL 300 ng/μL RNA and mixed completely. Meanwhile, the control group was carried out: Ten milligrams precipitated sepiolite was added to 10 μL 400 mM Guanidine Hydrochloride (pH 4.0) and mixed completely. The supernatant was transferred into a 200-μL microtube after centrifugation. The DNA was precipitated with 75% ethanol and dissolved in 5 μL TE. All the samples were checked using 1% agarose gel electrophoresis.

The effect of the addition of RNA to the plasmid transformation was performed as following: 50 μL of transforming mixture consisting of 50 μL LB, bacteria (OD_600 nm_ = 20), 1% sepiolite, 300 ng (or 3 μg) RNA and 100 ng pET15b were transferred into a 500-μL microtube (DNA is added in the end); the mixture was resuspended in a vortex mixer by mixing at full speed for 1 min; and then the cells were spread on a 1% agar plate with 100 μg/mL ampicillin and incubated at 37 °C overnight.

Finally, the effect of the degree of richness of the sepiolite fibers on the plasmid transformation was considered. For this purpose, 500 mg of sepiolite was suspended in 50 mL sterile water and mixed thoroughly. The sepiolite suspension was placed at room temperature for 2 h, and the supernatant was transferred into a new tube and collected through centrifugation at 5,000 × g for 2 min. The precipitation was suspended in 25 mL sterile water as sepiolite 2. Two hundred milligrams of sepiolite was suspended in 20 mL sterile water and ultrasonicated at 200 W for 2 min. The ultrasonicated sepiolite was regarded as sepiolite 3 and untreated sepiolite was named as sepiolite 1. Finally, 1 mg/mL of GFPuv was added to the sepiolite 1, 2 and 3 and mixed thoroughly. After absorbing GFPuv, the shape of the sepiolite was observed under a fluorescence microscope (Nikon TE2000, Nikon Corporation, Tokyo, Japan). Using the three kinds of sepiolites, plasmid transformation was performed as above mentioned and compared.

## 4. Conclusions

Using nanomaterials—based on the Yoshida effect—can make DNA transformation rapid and simple. However, the amount of 0.01% nanomaterials (w/v) presented previously is too low to be successful for bacterial transformation. We used 0.1% nanomaterials (w/v) to achieve enhanced transformation efficiency by approximately 100-fold. Based on the new result, vortex operation would be better than streaking a plate in a single continuous movement to further enhance the transformation up to almost 10-fold. Meanwhile, for such a transformation, there may be an alternative mechanism to explain our findings.
